# Stem cells and discogenic back pain

**DOI:** 10.1093/bmb/ldad008

**Published:** 2023-05-10

**Authors:** Luca Miranda, Marco Quaranta, Francesco Oliva, Nicola Maffulli

**Affiliations:** Department of Musculoskeletal Disorders, Faculty of Medicine and Surgery, University of Salerno, Via Salvador Allende, 43, Baronissi SA 84081, Italy; Clinica Ortopedica, Ospedale San Giovanni di Dio e Ruggi D’Aragona, Via San Leonardo, Salerno 84131, Italy; Department of Musculoskeletal Disorders, Faculty of Medicine and Surgery, University of Salerno, Via Salvador Allende, 43, Baronissi SA 84081, Italy; Clinica Ortopedica, Ospedale San Giovanni di Dio e Ruggi D’Aragona, Via San Leonardo, Salerno 84131, Italy; Department of Musculoskeletal Disorders, Faculty of Medicine and Surgery, University of Salerno, Via Salvador Allende, 43, Baronissi SA 84081, Italy; Clinica Ortopedica, Ospedale San Giovanni di Dio e Ruggi D’Aragona, Via San Leonardo, Salerno 84131, Italy; Department of Musculoskeletal Disorders, Faculty of Medicine and Surgery, University of Salerno, Via Salvador Allende, 43, Baronissi SA 84081, Italy; Clinica Ortopedica, Ospedale San Giovanni di Dio e Ruggi D’Aragona, Via San Leonardo, Salerno 84131, Italy; Centre for Sports and Exercise Medicine, Barts and the London School of Medicine and Dentistry, Queen Mary University of London, Mile End Hospital, 275 Bancroft Road, London E1 4DG, England; Guy Hilton Research Centre, Faculty of Medicine, School of Pharmacy and Bioengineering, Keele University, Thornburrow Drive, Hartshill, Stoke-on-Trent ST4 7QB, England

**Keywords:** mesenchymal stem cells, bone marrow, back pain, intradiscal injection

## Abstract

**Background:**

Chronic low back pain, common from the sixth decade, negatively impacts the quality of life of patients and health care systems. Recently, mesenchymal stem cells (MSCs) have been introduced in the management of degenerative discogenic pain. The present study summarizes the current knowledge on the effectiveness of MSCs in patients with discogenic back pain.

**Sources of data:**

We performed a systematic review of the literature following the PRISMA guidelines. We searched PubMed and Google Scholar database, and identified 14 articles about management of chronic low back pain with MSCs injection therapy. We recorded information on type of stem cells employed, culture medium, clinical scores and MRI outcomes.

**Areas of agreement:**

We identified a total of 303 patients. Ten studies used bone marrow stem cells. In the other four studies, different stem cells were used (of adipose, umbilical, or chondrocytic origin and a pre-packaged product). The most commonly used scores were Visual Analogue Scale and Oswestry Disability Index.

**Areas of controversy:**

There are few studies with many missing data.

**Growing points:**

The studies analysed demonstrate that intradiscal injections of MSCs are effective on discogenic low-back pain. This effect may result from inhibition of nociceptors, reduction of catabolism and repair of injured or degenerated tissues.

**Areas timely for developing research:**

Further research should define the most effective procedure, trying to standardize a single method.

## Introduction

Chronic low back pain is extremely common and mainly affects patients over 60, with a prevalence of about 70%,^1–4^ worsening the quality of life of patients and imposing negative economic consequences on health care systems.^1^^,^^5^

Recently, biological therapy with mesenchymal stem cells (MSCs) has been introduced in the management of discogenic pain and degenerative disc disease (DDD).^6^

Back pain of discogenic origin has a multifactorial pathogenesis, and genetic factors, age, body mass index (BMI), smoking, work activity and trauma contribute to the development of the pathology.^7–15^

Aging is accompanied by profound modifications of the intervertebral disc, including alterations of the normal anabolic/catabolic balance, which normally keeps the intervertebral disc intact.^16^ The nucleus pulposus loses water, and calcific areas induce a lower capacity to distribute load with a reduction of the intervertebral space.^13^^,^^17^ The lower synthesis of type I collagen, the main constituent of the fibrous annulus, progressively reduces the elastic properties of the nucleus pulposus, favouring protrusion and herniation.^18–21^ In addition to collagen, age-related changes also affect proteoglycans and the extra-cellular matrix (ECM). Generally, the ratio of chondroitin sulphate to keratan sulphate is in favour of the former; with age, this ratio is reversed, reducing hydrophilia.^22–24^

The metalloproteinases of the ECM are less subject to inhibitory control; in addition, degenerative processes induce an acidic environment that further promotes the activation of these enzymes, which participate in the degenerative processes of the disc.^25^ All the alterations to the disc, together with the continuous mechanical stresses to which the spine is subjected, affect the adjacent nerve structures and manifest with the appearance of pain.^26^ A high BMI increases the load on the discs, with possible earlier onset of discogenic pain.^27^

The management of discogenic low back pain can be conservative or surgical.^28^ Generally, the initial approach is conservative and includes nonsteroidal anti-inflammatory drugs (NSAIDs), muscle relaxants, opioids and physiotherapy.^29^^,^^30^

In most patients, conservative management should be attempted before surgical treatment, as local and systemic complications may occur following surgery, including deep vein thrombosis, infection and myocardial infarcts.^31–33^

In spinal fusion, for example, in addition to the risks of non-union and hardware failure, alterations to the adjacent upper and lower vertebral segments are common due to abnormal load distribution.^34^

Recently, stem cell therapies have been increasingly studied to promote regeneration of the disc structures that determine the onset of symptoms. Degenerative discopathy seems to be responsible for 40% of low back pain.^35^

The intervertebral disc has its own multipotent stem cells, with progenitor cells both in the nucleus pulposus and the annulus fibrosus, with markers typical of MSCs.^36^

These stem cells can differentiate and participate in regenerative processes.^37–40^ With age, these cells progressively reduce, affecting the repair capabilities of the intervertebral disc. In the annulus fibrosus, progenitor cells can differentiate into different cell lines, such as adipocytes, chondrocytes, osteoblasts and endothelial cells.^41^

Other stem cells, both adipose and medullary, can differentiate into cells with characteristics similar to those of the nucleus pulposus under appropriate stimuli.^42–44^  *In vitro*, inoculated MSCs can develop phenotypic features similar to the disc own cells, capable of synthesizing the different matrix components when stimulated by growth factors such as Transforming Growth Factor-β (TGF-β),^45–47^ growth differentiation factor 5 (GDF5) and growth differentiation factor 6 (GDF6) belonging to the TGF family. In these studies, GDF-5 favoured the phenotypic differentiation of bone marrow (BM) stem cells into cells of the nucleus pulposus by promoting the synthesis of type II collagen,^48^ but it did not stimulate the production of proteoglycans, as TGF-β1 did.^49–51^

Therefore, in stem cell therapy, it is important to consider both the type of stem cells and the growth factors used in combination with them, as well as the use of scaffolds.

Patients in whom stem cell therapy would be indicated present with early disc degeneration and mild to moderate pain, and failure of conservative therapy. Ideal patients are those with degenerative involvement of a single Pfirrmann Grade III–IV disc.^52^

This review defines the current knowledge on the effectiveness of biological therapy using MSCs in patients with discogenic back pain.

## Methods

**Fig. 1:**
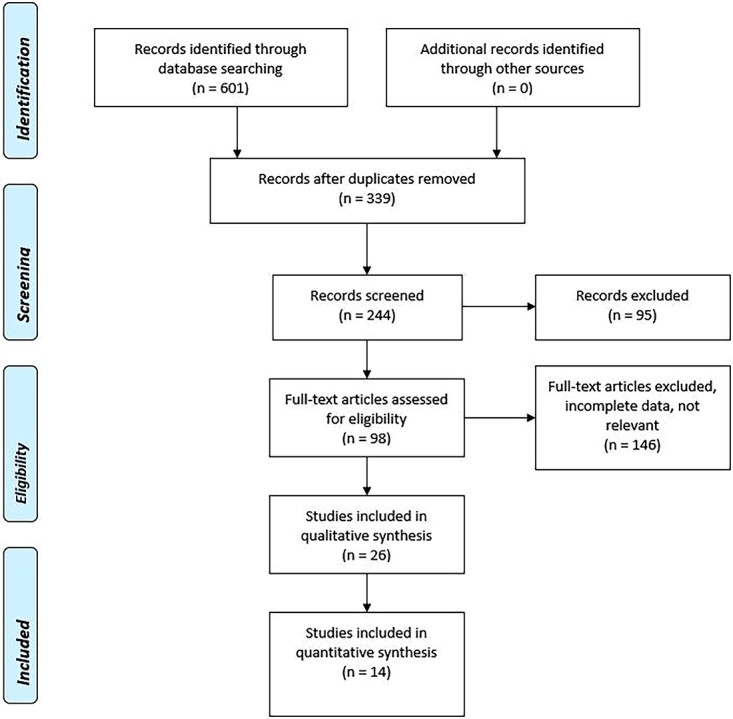
Correlation between MCMS and year of publication.

This study and its procedures were organized, conducted and reported following the Preferred Reporting Items for Systematic Reviews and Meta-Analyses (PRISMA) guidelines^53^ (Fig. 1).

### Eligibility criteria

We searched studies about the use of stem cells in the management of discogenic back pain. Studies included in the search are case reports and case series, clinical trials and systematic reviews. We excluded animal studies, editorials, narrative reviews and articles in which stem cells were used in combination with confounding factors that could affect the outcome such as PRP.

### Data sources and search

We performed an exhaustive search of all databases associated with PubMed and Scopus up to April 2023, using the following key words: MSCs, stem cells, back pain, discogenic back pain, intervertebral disc degeneration.

### Study selection

The articles resulting from the search were evaluated independently by two orthopaedic residents. A researcher experienced in systematic review solved cases of doubt. The initial selection of articles was based on the title and reading of the abstract. In accordance with inclusion and exclusion criteria previously reported, the articles considered relevant to the aim of the study were selected. Subsequently, these articles were read in their entirety to ascertain their actual relevance to the purposes of this review.

### Data collection

The data extracted from reading the articles included in the present systematic review were collated in an Excel database. Doubts and inconsistencies were followed and solved by discussion. The features analysed include:

- Type of stem cells employed
- Characteristics of the culture medium
- Clinical scores
- MRI outcomes

### Methodological assessment

We used the Modified Coleman Methodology Score (MCMS)^54^ criteria to assess the studies reviewed (Table 1). A score from 0 to 100 is assigned to each study; a score of 100 indicates a study in which there are no confounding factors or bias. The MCMS was correlated with publication year to examine the chronological trend in methodology.^54^

**Table 1:** MCMS

		Score
Part A: Only one score to be given for each of the 7 sections		
1. **Study size: number of patients**		
<30		0
30–50		4
50–100		7
>100		10
2. **Mean follow-up**		
<12 months		0
12–36 months		4
37–60 months		7
>61 months		10
3. **Surgical approach**		
Different approach used and outcome not reported separately		0
Different approach used and outcome reported separately		7
Single approach used		10
4. **Type of study**		
Retrospective cohort study		0
Prospective cohort study		10
Randomized controlled trial		15
5. **Description of diagnosis**		
Described without percentage specified		0
Described with percentage specified		5
6. **Description of surgical technique**		
Inadequate (not stated, unclear)		0
Fair (technique only stated)		5
Adequate (technique stated, details of surgical procedure given)		10
7. **Description of postoperative rehabilitation**		
Described		5
Not described		0
Part B: Scores may be given for each option in each of the 3 sections if applicable		
1. **Outcome criteria**		
Outcome measures clearly defined		2
Timing of outcome assessment clearly stated		2
Use of outcome criteria that has reported reliability		3
General health measure included		3
2. **Procedure of assessing outcomes**		
Participants recruited		5
Investigator independent of surgeon		4
Written assessment		3
Completion of assessment by patients themselves with minimal investigator assistance		33
3. **Description of subject selection process**		
Selection criteria reported and unbiased		5
Recruitment rate reported		
>90%		5
<90%		0

## Results

The initial search produced a total of 601 articles. After removal of duplicates, we obtained 339 articles. After the first abstract and title analysis, we excluded 95 articles. From the 244 remaining articles, we excluded 146 articles after full-text assessment. A total of 14 articles were included in the present review (Table 2).

**Table 2:** Studies included and main features

Study	Year of publication	Type of study	No. of patients	Stem Cells	Conclusions
Lewandrowski et al.^55^	2023	Retrospective	33	Allogenic BM MSCs	The injection of allogeneic MSCs to treat patients with painful intermediate-stage degenerative disc disease has merit.
Amirdelfan et al.^56^	2021	Randomized Controlled Trial	100	Allogenic BM MSCs	Intradiscal injection of MPCs could be a safe, effective, durable and minimally invasive therapy for subjects who have CLBP associated with moderate DDD.
Wolff et al.^57^	2020	Retrospective	33	Autologous Bone Marrow Concentrate	Autologous BMCs are a logical strategy to alleviate discogenic pain and restore patient function with the goal of providing a restorative therapy, which provides long-term benefits of reduced pain and improved disc health and function
Ju et al*.*^58^	2020	Randomized Controlled Trial	13	Cell-based stem cell treatment (MPC-06-ID, Mesoblast)	No difference in outcomes between therapeutic intradiscal agents and the control saline arm. In all groups, patient reported pain and disability scores decreased significantly
Centeno et al*.*^59^	2017	Pilot study	33	Autologous Bone Marrow Concentrate	The intradiscal injection of culture expanded MSCs to treat DDD with symptomatic disc bulge produced encouraging results: reduced pain, increased function and reduced disc bulge size in most patients
Kumar et al.^60^	2017	Single-arm clinical trial	10	AT-MSCs	The study confirmed the safety and tolerability of coinjection of AT-MSCs and a HA derivative in patients with intervertebral disc degeneration
Pettine et al.^61^	2017	Prospective, open-label, non-randomized, single-arm study	26	Autologous Bone Marrow Concentrate	These results indicate that injection of BM concentrate has the potential to provide a non-surgical option for patients with chronic discogenic low back pain
Elabd et al.^62^	2016	Case Series	5	Autologous BM MSCs	Intra-discal injection of autologous, hypoxic cultured BM-derived MSCs demonstrated safety and feasibility in five patients diagnosed with DDD.
Noriega et al.^63^	2016	Randomized Controlled Trial	12	Allogenic BM MSCs	The therapy with expanded allogeneic BM-derived MSC results in significant relief of pain and disability, and quantitative MRI evidence suggests partial disc healing. The healing effects appear to be smaller than those reported for treatment with autologous MSC.
Mochida et al.^64^	2015	Prospective Clinical Study	9	NP cells co-cultured in direct contact with autologous BMA-MSCs.	The study confirmed the safety of activated NP-cell transplantation and provided promising findings that suggest the minimal efficacy of this treatment to slow the further degeneration of human intervertebral discs
Pang et al.^65^	2014	Clinical Trial	2	Human umbilical cord tissue-derived mesenchymal stem cells (HUC-MSCs)	The study indicates that HUC-MSC transplantation is a favourable alternative method for the treatment of chronic discogenic low back pain.
Coric et al.^52^	2013	Prospective Study	15	NuQu® allogeneic juvenile chondrocytes (ISTO Technologies)	Preliminary safety was demonstrated, and clinical results were encouraging, with statistically significant improvements in ODI, NRS and SF-36 scores
Orozco et al.^66^	2011	Clinical Trial	10	Autologous BM MSCs	The therapy with BM-derived MSCs may be a valid alternative treatment for chronic back pain caused by DDD. Advantages over current gold standards include simpler and more conservative intervention without surgery, preservation of normal biomechanics and same or better pain relief
Yoshikawa et al.^67^	2010	Case Report	2	Autologous BM MSCs	The intervertebral disc regeneration therapy using MSC brought about favourable results. It seems to be a promising minimally invasive treatment

Ten of 14 studies used stem cells derived from the BM. Three of these studies used Bone Marrow Concentrate. In one of these studies, stem cells were cultured next to the nucleus pulpous (NP). In the remaining four studies, different stem cells were used [adipose, umbilical, chondrocytarian origin (NuQu® allogeneic juvenile chondrocytes)] and a pre-packaged product, Mesoblast (MPC-06-ID, Mesoblast), was also employed.

The details of the culture are reported in Table 3.

**Table 3:** Injection characteristics

Study	Stem cells	Media	Injected solution	Injection site	Injection volume
Lewandrowski et al.^55^	BM allogenic	Dulbecco’s modified eagle medium (DMEM)	Hyaluronic acid derived from immunoselected umbilical cord stem cells	Intradiscal	2 ml
Amirdelfan et al.^56^	BMA	–	Hyaluronic acid	Intradiscal	2 ml
Wolff et al.^57^	BMC	Magellan Autologous Platelet Separator System (Isto Biologics, Hopkinton	MSCs, platelets, growth factors.	Intradiscal	3 ml
Ju et al*.*^58^	Mesoblast	–	Hyaluronic acid	Intradiscal	2 ml
Centeno et al*.*^59^	BMC	Platelet lysate; Doxycycline; Heparin; Hypoxic conditions	Platelet lysate	Intradiscal	–
Kumar et al.^60^	AT-MSCs	–	1% Hyaluronic Acid Tissuefill® (HA derivative; CHA Meditech Co., Ltd, Daejeon, South Korea)	Intradiscal	2 ml
Pettine et al.^61^	BMC	ART BMC (Celling Biosciences, Austin, TX)	–	Intradiscal	2–3 ml
Elabd et al.^62^	BMA	Dulbecco’s modified eagle medium (DMEM) with 10% platelet lysate, 5 μg/ml doxycycline, and 2 IU/ml heparin in a 37°C/5% CO_2_/5% O_2_ incubator (hypoxic conditions)	BMA + autologous platelet lysate	Intradiscal	0.25–1 ml
Noriega et al.^63^	BM allogenic	Ringer Lactate	Saline solution	Intradiscal	2 ml
Mochida et al.^64^	BMA	Serum; NP cells	Saline solution	Intradiscal	1 ml
Pang et al.^65^	HUC-MSCs	Dulbecco’s modified Eagle’s medium (DMEM, Gibco) + 10% FBS	–	Intradiscal	1 ml
Coric et al.^52^	NuQu® allogeneic juvenile chondrocytes	Gentamicin, l-glutamine, growth factors, and l-ascorbate	Fibrin	Intradiscal	1–2 ml
Orozco et al.^66^	BMA	–	–	Intradiscal	–
Yoshikawa et al.^67^	BMA	15% autologous serum, gentamicin; 100 nmol/L estriol; 0.1% trypsin	Collagen sponge	Intradiscal	10 ml

Stem cells were mixed with other substances before injection. In three studies, a platelet lysate was used; in two, a saline solution; in four, hyaluronic acid; in one, fibrin; in 1one collagen sponges were used. The injection volume varied between 1 and 3 ml. Yoshikawa et al. used collagen sponges with a volume of 10 ml.^67^

All studies reported beneficial results of stem cell therapy, with improvements in pain, strength and return to daily and work activities.

Different scores were used. The most commonly used are VAS and ODI, used in 9 of 14 and 10 of 14 studies, respectively. Other scores were: SF-36, used in 5 of 14 studies; NRS, in 2 of 14; JOA, in 2 of 14. In relation to the VAS, 5 of 8 studies used a scale from 0 to 100, 2 of 8 from 0 to 10 and 1 study did not report such data.

Using the *t* student between ODI pre and post management, the *P*-value is 0.0004; similarly for the VAS score, the *P*-value is <0.0001.

The details of the different scores are reported in Table 4.

**Table 4:** Scores

Study	Scores	Pre-treatment	Post-treatment
Lewandrowski et al.^55^	VAS ODI	8.2 44.8	1.74 6.07
Amirdelfan et al.^56^	VAS ODI SF-36 WPAI	70 48.5––	––––
Wolff et al.^57^	ODI NRS SF-36	36.7 5.2 53.4	–––
Ju et al*.*^58^	VAS ODI	66.5 20	21.8 9.3
Centeno et al*.*^59^	SANE NPS FRI	– 5.2 60.5	53 3.3 30
Kumar et al.^60^	VAS ODI SF-36	6.5 42.8	2.9 16.8
Pettine et al.^61^	VAS ODI	82.1 56.7	21.9 17.5
Elabd et al.^62^	–	–	–
Noriega et al.^63^	VAS ODI SF-12 men SF-12 phy	67 34 46 39	47 22 48 45
Mochida et al.^64^	JOA LBP	14.2 1.2	27.2 2.7
Pang et al.^65^	VAS ODI	7.5 51	2.5 12.5
Coric et al.^52^	ODI NRS SF-36 men SF-36 phy	53.1 35.2 48.5 35.5	20.3 3.1 50.5 46.9
Orozco et al.^66^	VAS ODI SF-36 men SF-36 phy	68.9 25 54.1 12.7	20 7.4 49.7 24.8
Yoshikawa et al.^67^	JOA VAS	2.5 –	17 –

The MRI baseline characteristics of early stage patients were disc hydration, height, bulging or protrusions and annulus tears. The MRI was repeated at follow-up to identify any changes in these characteristics. In 7 of 14, studies, the water content of the disc was evaluated with the MRI T2-weighted sequence, evidencing that hydration had increased. The height of the disc was assessed in 8 of 14 studies, with encouraging results related to the conservation or increase of the height of the discs. Bulging was evaluated in 4 of 14 studies, with a reduction in at least 23% of cases. In 6 of 13 studies, the condition of the spine was graded using Modic criteria, from grade I to III; 6 of 14 studies used the Pfirrmann grading system, from grade I to V; finally in 2 of 14 studies the Modified Dallas Discogram Description from a grade 0 to IV was used.

In 3 of 14 studies, the most common adverse effect was injection pain, treated with NSAIDs and opioids. The use of subsequent surgical treatment was considered as failure of stem cell therapy; this occurred in 4 of 303 patients.

### MCMSs

**Fig. 2:**
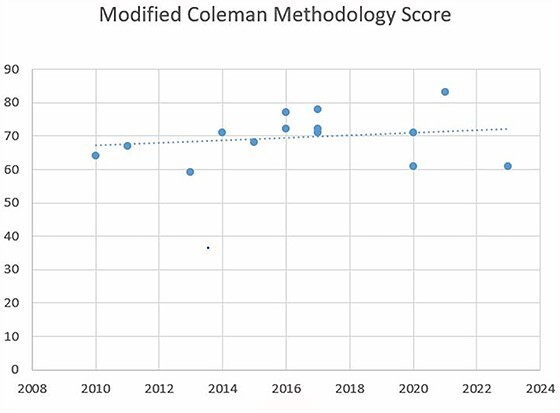
PRISMA flow diagram 2009.

Calculating the Pearson’s correlation coefficient between MCMS and the year of publication (Fig. 2), we obtained a positive association (*r* = 0.48, *P*-value 0.1). In recent years, there was not an improvement in methodology.

The mean MCMS score was 69.64. Table 5 reports mean, SD and range for each MCMS criteria.

**Table 5:** Mean score for each MCMS criteria

Methodology criterion	Mean score (SD)	Range
*Part A*		
1. Study size	1.64 (2.4)	0–7
2. Follow-up	2.35 (3.2)	0–10
3. N procedures	10 (0)	10
4. Type of study	10 (4.8)	0–15
5. Diagnostic certainly	5 (0)	5
6. Description of surgical technique	9.28 (1.81)	5–10
7. Rehabilitation & compliance	0.35 (1.33)	0–5
*Part B*		
1. Outcome criteria	9.35 (1.27)	7–10
2. Outcome assessment	12.0 (0)	12
3. Selection process	9.64 (1.33)	5–10
MCMS	69.64 (6.92)	59–83

## Discussion

MSCs have been used for regenerative therapy in different musculo-skeletal conditions. MSCs have been shown to be effective and safe in osteoarthritis and meniscal, and tendon and ligament injuries.^68^ MSC can be obtained from different tissues: fat, BM and umbilical cord. Stem cells derived from the BM are the most commonly studied, although stem cells derived from adipose tissue are more numerous. Adipose tissue-derived mesenchymal stem cells (AT-MSCs) have a lower capability to differentiate in chondrocytes; in some studies, preculture with NP cells was performed to increase their regenerative capability.^69^ Stem cells derived from the umbilical cord are used for their low immunogenicity. Discogenic back pain is one of the most common conditions affecting individuals between the fifth and seventh decade, and it is estimated that in 2050 over 2 billion people will be over 60.^70^ There is no association between pain and MRI appearance.^71^

During the progression of this chronic condition, there is a shift from type I to type II collagen with progressive dehydration of the ECM and consequent reduction of the mechanical support capability of the disc.^16^

Cell transplant therapy, involving both MSC and NP, has resulted in increased water content in the disc and consequent height restoration in both *in vivo* and human studies.^36^^,^^72–74^ The percutaneous implantation of MSC may induce pain relief with three mechanisms: inhibition of nociceptors, reduction of catabolism and repair of tissues. Noriega et al.^75^ used stem cells derived from allogeneic marrow without adverse events. They quantified the slope of pain relief from baseline to compare between the various trials, and an efficiency of allogeneic of 0.28 versus autologous MSCs of 0.71 was documented. Some studies used NP cells to prevent ‘*graft versus host disease*’, but these cells had a poor capacity for ECM regeneration.^64^ Mochida et al.^64^ cultured NP cells together with MSC to increase the synthetic capacity of autologous NP cells and reduce the risk of GVHD. Umbilical MSCs could differentiate into NP when cultured with them.^76^

Coric et al.^52^ used allogeneic chondrocytes to avoid damage to the already damaged NP, further aggravating the pathology. Cells from young patients showed a greater ability to synthesize ECM, without causing GVHD.

One aspect to consider is the low-oxygen environment of the disc, which is also required for successful MSCs culture. Indeed, cells grown at normal oxygen concentrations induced an increase in disc hydration, but not in height.^66^ Several studies reported on the cross-talk between the injected MSC and the native NP cells, in particular the TGF-beta signalling system, hypothesizing a major role in the regeneration of ECM.^77^

Overall the studies included in this review indicate that percutaneous injection of intradiscal MSC was safe and resulted in a high success rate.

A multicentre study^58^ evaluated four types of therapies (Growth factor BMP-7, Active fibrin sealant, Growth factor rhGDF-5, MSC), comparing them to placebo (saline solution) and obtaining good results. A possible effect of the injection of saline solution is the dilution of the cytokines responsible for inflammation.^78^

Noriega et al.^63^ obtained interesting results in relation to the time of follow-up. In the control group, which received an injection of local anaesthetic, they obtained a decrease of VAS within 8 days from the administration, without further improvements; the ODI worsened during the year of follow-up. Instead, in the study group with MSC, the greatest effect was achieved at about 3 months and maintained at 6 and 12 months follow-up.

Different scores were used in the various studies to evaluate the state of degeneration of the disc and, consequently, the eligibility of patients for therapy. Patients with complete annular fissuration could not be treated because of disc incontinence. During the injection, Kumar et al.^60^ suspended the MSCs with a derivative hyaluronic acid, aiming to reduce or prevent the dispersion of stem cells and any differentiation in osteoblasts.

MSCs can differentiate into fibroblasts^59^ and strengthen the annulus, preventing herniation by depositing new collagen fibres. In fact, 85% of patients showed a reduction in posterior bulge. A reduction of at least 25% of the bulge decreased the pain significantly. Only one case of herniation that required surgery was reported after 5 months. This complication could have resulted from needle injection, excessive proliferation of MSCs or excessive production of ECM.

Among the complications related to the injection of MSCs is the formation of osteophytes in the tissues surrounding the injection site.^79^

When conservative therapy failed, it is possible to use different surgical methods,^7^^,^^80^ but these have several complications: dural lesions, infections and epidural hematomas.^81–84^ In stabilization of the spine, for example, by limiting the movements of the affected section of the spine, the stress imposed to the adjacent vertebrae is increased, contributing to the degeneration of those discs. Pettine et al. reported about the reduced length of hospital stay with MSC compared with surgical treatment, which involves 5 days in hospital.^61^ Despite this, some failure necessitated surgical treatment; for example, three patients were treated surgically between 6 and 12 months after implantation of MSC for persistent pain.^52^

New therapeutic approaches aim to induce the migration of MSCs to the damaged site and warrant further exploration.^85^^,^^86^

The limitations of this study are related to the low number of articles, the lack of data on patients, the aetiology of discogenic back pain, the type of culture medium and the solution injected, and the use of different clinical scores in the various studies. All these do not allow to obtain homogeneous results regarding treatment efficacy.

## Conclusion

Stem cells are a promising potential resource to be exploited in the management of musculoskeletal conditions associated with aging, in which the cellular regenerative capabilities can be employed. Further research efforts should define the actual effectiveness of MSCs in the different areas of their use.

## Data Availability

All data generated or analysed during this study are included in this published article.
